# Predictive Maintenance in Building Facilities: A Machine Learning-Based Approach

**DOI:** 10.3390/s21041044

**Published:** 2021-02-03

**Authors:** Yassine Bouabdallaoui, Zoubeir Lafhaj, Pascal Yim, Laure Ducoulombier, Belkacem Bennadji

**Affiliations:** 1 Laboratoire de Mécanique Multiphysique Multiéchelle, LaMcube, UMR 9013, Centrale Lille, CNRS, Université de Lille, F-59000 Lille, France; zoubeir.lafhaj@centralelille.fr; 2 Centre de Recherche en Informatique Signal et Automatique de Lille, CRIStAL, UMR 9189, Centrale Lille, CNRS, Université de Lille, F-59000 Lille, France; pascal.yim@centralelille.fr; 3 Bouygues Construction, 78280 Guyancourt, France; l.ducoulombier@bouygues-construction.com; 4 Bouygues Energies et Services, 78280 Guyancourt, France; b.bennadji@bouygues-es.com

**Keywords:** predictive maintenance, buildings, IoT, data, machine learning, autoencoders, HVAC

## Abstract

The operation and maintenance of buildings has seen several advances in recent years. Multiple information and communication technology (ICT) solutions have been introduced to better manage building maintenance. However, maintenance practices in buildings remain less efficient and lead to significant energy waste. In this paper, a predictive maintenance framework based on machine learning techniques is proposed. This framework aims to provide guidelines to implement predictive maintenance for building installations. The framework is organised into five steps: data collection, data processing, model development, fault notification and model improvement. A sport facility was selected as a case study in this work to demonstrate the framework. Data were collected from different heating ventilation and air conditioning (HVAC) installations using Internet of Things (IoT) devices and a building automation system (BAS). Then, a deep learning model was used to predict failures. The case study showed the potential of this framework to predict failures. However, multiple obstacles and barriers were observed related to data availability and feedback collection. The overall results of this paper can help to provide guidelines for scientists and practitioners to implement predictive maintenance approaches in buildings.

## 1. Introduction

In Europe, buildings are responsible for 40% of energy consumption, and approximately 28% of total direct and indirect CO_2_ emissions [1,2]. A large part of this energy is consumed by building installations, mainly cooling and heating systems. Indeed, faulty operations of building installations lead to significant waste, causing up to a 20–30% increase in the total building energy consumption [3]. To achieve energy efficiency goals, building maintenance and management approaches must be improved and optimised.

Facility management (FM) teams depend on real-time, accurate and comprehensive data to perform day-to-day maintenance activities and to provide accurate information to top management [4]. However, the activities of inspecting facilities, assessing maintenance and collecting data are labour-intensive and time consuming [5]. In addition, the budget and resources allocated for building maintenance are limited [6], and maintenance personnel argue that their budget and resources are insufficient and below their needs [7,8]. This trade-off affects the quality and the relevance of the maintenance activities and inspections, which leads to poor maintenance and quality management policies in facilities.

Nowadays, maintenance practices in the field of FM are mainly based on corrective maintenance: late actions are taken following a user complaint or an unplanned failure [9]. The lack of a budget and human resources limits the use of preventive maintenance to the minimal level of mandatory inspections in critical installations. Moreover, the concept of predictive maintenance is based on processing the operation data sent via sensors using data analytics techniques. This approach offers the possibility to address the gap in maintenance practices by supporting the FM teams to take early action and avoid unplanned failures without the need for costly intensive site inspections of the installations.

Recently machine learning and data science techniques have been used in many aspects of modern society, including the Internet, finance, insurance, and medical applications [10]. In addition to the recent advances in machine learning, the introduction of smart devices and the Internet of Things allowed for the connection of physical assets and real-time data streaming at a low cost [11,12]. As a consequence, the field of operation and maintenance management is evolving. Classic maintenance approaches and quality management methods that were all controlled by people in the past are being transformed into predictive maintenance thanks to machine learning (ML) and artificial intelligence (AI) [13]. The industry of facility management can benefit from the rise of machine learning and IoT in order to improve the management of their assets and reduce waste. This paper presents a predictive maintenance approach for the field of facility management; for this purpose, a framework based on machine learning is proposed whilst taking into account the specificities of the FM field.

## 2. Research Background

This section first presents an overview of the main maintenance approaches and describes the differences between them. Following this, an overview of the deep learning algorithms used in this research is presented: autoencoders, recurrent neural networks and long short-term memory (LSTM).

### 2.1. Maintenance Approches

According to the British standards [14], maintenance is the combination of all technical, administrative, and managerial actions during the life cycle of an item intended to retain it in or restore it to a state in which it can perform the required function. Approaches of maintenance management can be grouped into three main groups as follows [15]:

- Corrective maintenance: also known as reactive maintenance or run to failure maintenance, which consists of intervening after the failure. The equipment is allowed to operate until it fails.
- Preventive maintenance: this consists of carrying out inspection and maintenance actions while the equipment is still running to reduce the probability of breakdowns. Preventive maintenance can be either time-based via a schedule or usage-based (e.g., every 100 km). This approach helps to reduce the number of failures, but unnecessary inspections are performed and unplanned failures still occur, which increases the cost of maintenance.
- Predictive maintenance: this approach is based on using condition monitoring data to predict the future machine health state [15]. This approach aims to predict when, where, and which components may have potential failures.

Nowadays, maintenance practices are mainly corrective and preventive; predictive maintenance is only applied for critical situations [16]. Traditionally in facility management, aside from mandatory tests and inspections for critical equipment such as boilers and chillers, the majority of maintenance activities in buildings remain mainly corrective, simply responding to users’ complaints or to unplanned failures [9].

Previous studies have addressed the amelioration of preventive maintenance in the FM field. The most important issue for the maintenance manager is to anticipate the suitable time for the effective implementation of each maintenance activity [17] within the limits of the maintenance budget and the available resources. With this intent, studies were conducted to optimise the inspection scheduling in HVAC installations using optimisation techniques such as the Monte Carlo method [18,19]. Similar studies used data mining techniques and time series forecasting to optimise the maintenance scheduling based on the history of the maintenance operations in the building [17]. These studies showed interesting results by optimising the inspection periods. However, unplanned failures still occurred. Different studies focused on adopting industrial maintenance techniques such as mechanical vibration analysis to monitor building installations using Fourier transformation and fuzzy logic [20,21] or simulation techniques for fault detection [22]; similarly, statistical models including linear and nonlinear regression were used for fault detection and diagnostics in HVAC units [23]. However, the high cost of the modelling and the simulation as well as the limitation to generalise the models on similar installations have limited the use of these techniques in the FM field. 

The predictive maintenance approach presents an opportunity for the FM sector to reduce unplanned failures, reduce maintenance costs and penalties, as well as to improve the comfort and the security of the inhabitants. However, implementing a predictive maintenance approach presents multiple challenges, such as connecting physical assets, extracting valuable data and developing accurate predictive algorithms. Indeed, the concept of predictive maintenance is not new; multiple studies were conducted in the past few decades, mainly focusing on statistical approaches [24]. Despite this, the deployment of effective predictive solutions remained expensive and difficult to implement. Furthermore, the recent development in the industry has increased machine complexity, which makes it difficult to predict failures with conventional methods [25]. Simultaneously, machine learning techniques have been gaining ground from computer vision [26] to natural language processing [27], from medical applications [28] to games [29], including applications in predictive maintenance and anomaly detection [30,31,32,33].

### 2.2. Deep Learning Overview

Deep learning is a sub-field of machine learning and an approach of artificial intelligence. It is a set of methods based on representation learning. It consists of representing the learning task as an embedded hierarchy of concepts to facilitate the extraction of useful patterns from raw data [10,34]. This hierarchy is represented as an artificial neural network of several layers, with each layer containing several neurons, and each neuron performing a simple but nonlinear transformation. The composition of these simple transformations allows for the learning of complex representations and solving complex tasks. Deep learning has accelerated the advancement of multiple aspects in modern society, from machine translation [35] to self-driving cars [36]. There are multiple deep learning models, and they vary in terms of design and architecture. Their use depends on the nature of the task (classification, prediction, clustering etc.) and the nature of the input data (text, images, sequences, etc.). A brief review of the deep learning architectures used in this work is presented below. 

#### 2.2.1. Autoencoders

**Figure 1:**
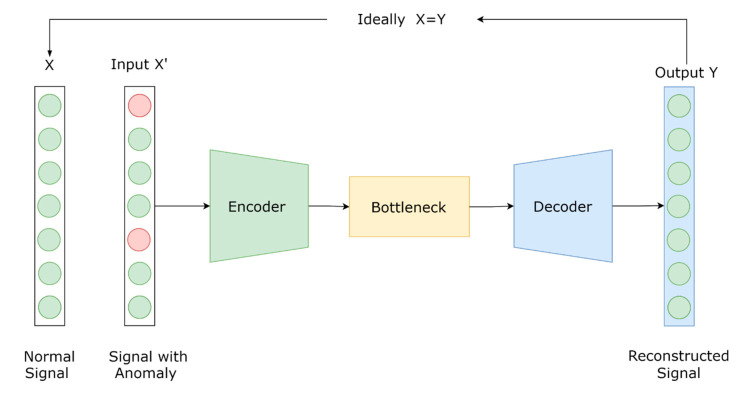
Illustration of an autoencoder architecture.

Autoencoders are a set of deep learning architectures; they can be considered as a special form of neural networks designed for unsupervised learning tasks [37]. The learning process is unsupervised since there is no label variable. In this type of neural network, the output variable is set to have the same dimension as the input variable [38]. An autoencoder is composed of two processes—an encoder and a decoder. The encoder transforms the input data trying to dig out hidden representations, while the decoder tries to reconstruct the input data from the hidden representations [37,39]. This process of encoding and decoding the input data can be seen as a learning circuit that tries to reconstruct the inputs with the minimum amount of distortion and noise [40]. An illustration of an autoencoder architecture is presented below in Figure 1. Autoencoders have been widely used for dimensionality reduction applications [41], signal reconstruction applications, and anomaly detection applications [37,42,43].

#### 2.2.2. Long Short-Term Memory (LSTM)

Long short-term memory is a variant of the artificial recurrent neural network (RNN) architecture, which is a type of artificial neural network designed to process sequential data as time series data and text and speech data [44]. LSTM and RNNs in general are capable of capturing long-term dependencies in a sequence. This means they can capture information about the past of the sequence. Thanks to this characteristic, LSTM are widely used in multiple applications including natural language processing applications [44], forecasting time series [35], and anomaly detection [30,45]. 

## 3. Research Objective and Methodology

### 3.1. Aim of the Current Study

The aim of this study is to propose a generic framework for predictive maintenance in order to reduce unplanned failures and minimise faulty operations in building installations. However, buildings are different, in terms of size, occupancy, and use. Thus, a generic framework should be flexible and adjustable to the differences between one building and another. For this reason, this study focuses on providing general guidelines to implement predictive maintenance. However, some propositions in this framework, such as the architecture of the predictive model or the collected data, can be changed depending on the building context and its environment. The objective is that the approach can be applied to any type of building installation (HVAC, lift, electrical machinery, etc.). Later in the study, this framework is tested using a case study of a sport facility.

### 3.2. Quantitative Research

Since the implementation of predictive maintenance and machine learning approaches in buildings is still new, there is little empirical research on this topic. To gather data and complete the literature research, the authors used face-to-face interviews with experts operating in facility management. Interviews were conducted with six experts. The goals of the interviews were: (1) to identify the actual tools used in the building environment, (2) to identify the available data, and (3) to assess their perspectives of the framework. The results of the interviews were used alongside the literature research to identify the list of the data sources in the building environment. These interviews helped us to design the fault detection and the feedback modules in the framework found in Section 4. Interviewees were asked questions such as: how do you (the user) expect the interface of the fault detection? Would you like to control the number of alerts? How would you like to formulate and send your feedback in the application? 

## 4. Framework Design

### 4.1. Defining Data Sources

The first step in this study was to define the data sources available in the building environment. Below, a list of data sources in the building environment are identified.

- Building automation systems (BAS): BAS are largely used in modern buildings to control and monitor the different installations via real-time data [46]. BAS contain both numerical and categorical data. Typical examples of numerical data are measurements such as temperature, energy consumption, and air and water flow rate, etc., whereas categorical data consist of time, alerts, and the binary state (ON/OFF), etc. [37]
- IoT devices and sensors: connected sensors and IoT devices have been introduced in buildings in recent years in order to collect information on the building and its surroundings [47,48,49]. These devices can be used to collect multiple types of information; they can be deployed on the installations (air handling unit (AHU), lift, chiller, etc.) to extract data such as temperature and vibration [50]. They can be used to collect human behaviour data such as occupation or mobility [48,51]. They can also be used to collect indoor and outdoor environmental measurements such as CO_2_ levels [52].
- Computerised maintenance management systems (CMMS): CMMS are used to manage daily maintenance activities. Functionalities of CMMS include: receiving emergency work orders and users’ requests, scheduling preventive maintenance activities, recording the history of maintenance activities, and inventory control, etc. [53,54]. Thus, CMMS represents an important data source for predictive maintenance.
- Building information modelling (BIM): the BIM model provides architectural 3D visualisation and standardisation of building information exchange between the stockholders along the construction project life cycle [55]. In recent years, several studies have been carried out to implement the BIM in the operation and maintenance phase [56,57,58]. BIM can be used to support FM teams while operating maintenance activities [59], to monitor energy efficiency in buildings [57] and to provide visual analytics for maintenance activities [60].
- Other data sources: building energy management system (BEMS), computer aided facility management (CAFM) and integrated workplace management system (IWMS) are examples of other data sources that can be found in the building environment. However, their use is limited to some specific facilities.

### 4.2. The Framework Architecture

**Figure 2:**
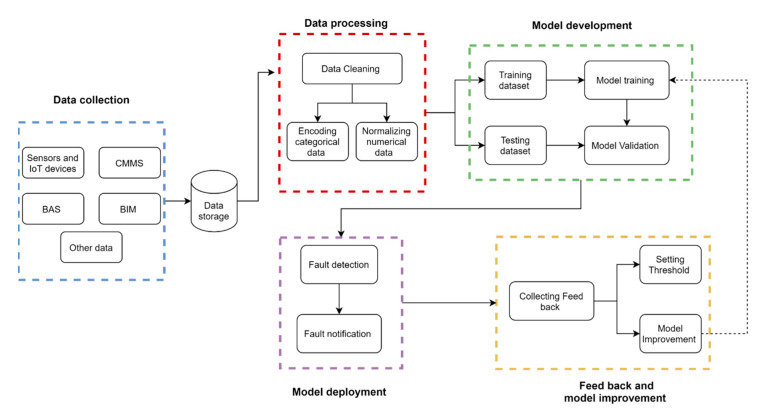
The proposed framework architecture.

The proposed framework in Figure 2 represents a machine learning approach adapted to the building context. The framework is composed of five steps: data collection, data processing, model development, fault notification and model improvement. All the steps are discussed and detailed below.

### 4.3. Data Collection, Storage and Processing

This part consists of preparing the data flow for the learning process. It starts with collecting data from the sources then storing it before applying necessary data cleaning and transformation.

- Data collection: the first step of the framework aims to collect data from the available sources in the building environment. It involves defining the data sources in the building then connecting them to extract the necessary data. The data sources were defined in this work in Section 4.1. However, data collection methods are not specified in this framework, since they depend on the user preference and the available ICT infrastructure.
- Data storage: this consists of storing the data after collecting them in a storage medium. There are different storage methods (cloud, local, etc.) which depend on the preference and the infrastructure of the user. in this framework, data storage is not discussed.
- Data pre-processing: the purpose of this step is to transform the raw data into a structured dataset ready for the training process. This step is comprised of two main parts:
  ○ Data cleaning: this consists of cleaning the data entries by removing irrelevant entries, replacing Nan values, and treating outliers.
  ○ Data transformation: in this study, only two transformations were proposed: normalisation of numerical features and encoding of categorical features. However, other transformations and feature engineering can be used [61,62].

### 4.4. Model Development

In this framework, the dataset is split into a training set and a testing set. The training set is used while training the model to learn the anomaly patterns, while the testing set is used to validate the model and to tune its parameters such as the anomaly threshold. In this study, an autoencoder architecture was chosen for the machine-learning model.

#### 4.4.1. The Autoencoder Model

**Figure 3:**
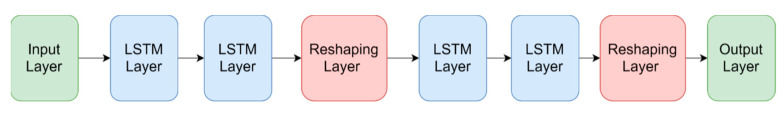
Illustration of the autoencoder model.

The proposed model has the architecture of an autoencoder: the encoder part consists of two LSTM layers followed by a reshaping layer to reshape the vector into the right dimension. The decoder part is similar and is composed of two LSTM layers and a reshaping layer. The resultant vector in the output layer has the same dimension as the input vector (Figure 3).

The choice of the autoencoder is justified by the model’s nature as an unsupervised learning algorithm [63]. The autoencoder does not require labelled datasets (data explicitly tagged with fault/normal labels) which are generally not available in buildings. Furthermore, autoencoders are flexible methods that require less hand engineering work and can be adapted to several applications. Moreover, the choice of LSTM layers is justified because they are designed to process sequential data such as time-series [44], which is the case of the majority of the installations monitoring data (temperature, vibration, and energy consumption, etc.). 

#### 4.4.2. Anomaly Score

Anomaly score is an evaluation metric to calculate the distance between the input vector X and the output vector $\hat{X}$. There are multiple evaluation metrics such as root mean square error (RMSE), mean absolute error (MAE), etc.

In this study, RMSE was used. It is defined as below (Equation (1)).

Let $X=\left(x_{1},x_{2},\ldots .,x_{n}\right)$ and $\hat{X}=\left(\hat{x_{1}},\hat{x_{2}},\ldots .,\hat{x_{n}}\right)$

(1) $$\mathrm{RMSE}\left(X,\hat{X}\right)=\sqrt{\frac{\sum_{1}^{n}{\left(x_{i}-\hat{x_{i}}\right)}^{2}}{n}}$$

**Figure 4:**
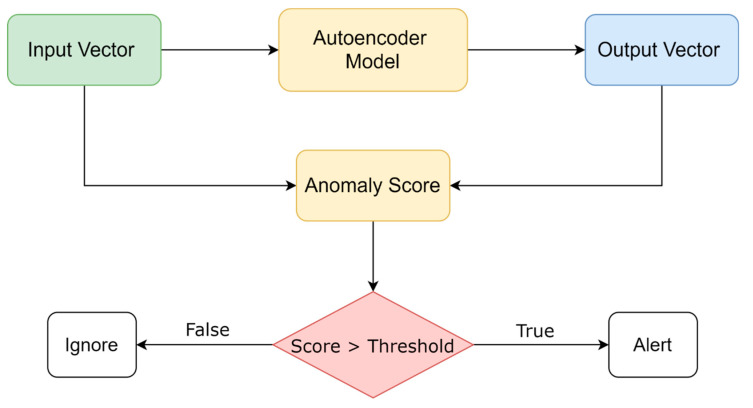
An Illustration of the process of calculating the anomaly score.

A threshold is defined depending on the training algorithm and depending on the targeted accuracy. If the anomaly score at a given time is higher than the defined threshold, the model sends an anomaly alert. This process is illustrated in Figure 4. 

### 4.5. Fault Notification

In this step, the model is deployed online after the training and the validation steps are done. Data are collected and processed before they are streamed to the fault detection model. If the anomaly score is bigger than the defined threshold, a notification is displayed in the CMMS then sent to the facility manager via SMS. The notification contains the name of the equipment, its location, and the time of the alert event. 

### 4.6. Feedback and Continuous Improvement

Machine learning models need to be regularly updated in order to improve the accuracy of the results. For this reason, collecting feedback after the deployment is crucial to improve the fault prediction model. The feedback is collected from the facility management team (the users) where they can report false alerts or undetected failures. This feedback is collected and stored via the CMMS. After the feedback is collected, a procedure of error evaluation is carried out, where the errors of the model are inspected. Following this, the model is updated using new training data, and its parameters are tuned to reduce the errors ratio. 

The improvement of the model is not a systematic approach; the procedure of updating the model using the collected feedback should be done via a proper schedule and by a machine learning specialist. In order to give the FM team a quicker response, the anomaly threshold was designed as an external parameter where the user can directly change the setting without a need for a total update to the model. 

### 4.7. Model Implementation

The framework can be applied to the different installations in the building including HVAC, lifts, and pumps. After defining the installations that will be involved, data are collected following the guidelines in Section 4.1; these data can also be completed by the use of IoT sensors such as vibration sensors, temperature sensors, and energy consumption meters, as illustrated later in the case study. As highlighted previously, the FM teams are free to choose how to send and store the data depending on the available ICT infrastructure in the building; for example, in the case study below, the Lora network was used to send the data that were stored and processed in the cloud. The first implementation of the model requires a period of collecting data to create the training and validation datasets. The development step in the framework is based on the classic ML validation methodology by splitting the dataset into a training set and a validation set. The proposed autoencoder model is flexible and can be deployed with different types of time series data. After the validation phase is completed, the model can be deployed online in the facility, the framework proposes to integrate the FM team in order to set the alert threshold depending on the availability of the team to check the alerts and on the criticality of each installation. The framework proposes integrating the FM team into the model improvement process. The FM team can continuously change the threshold alert to improve the accuracy of the model. Their feedback is also collected for future updates of the model. Since the predictive model is based on a deep learning model, the predictive model can be reused on similar installations in different facilities by using transfer learning [64], which can allow one to cut the development cost and reduce the implementation time.

## 5. Case Study: Predictive Maintenance for HVAC Installations in Sport Facility Buildings

### 5.1. The Facility Characteristics

The case study in this paper was conducted at a sport facility in the Paris region, France. It is composed of two principal buildings covering an area of 15,000 m^2^. The facility contains multiple installations. However, for accessibility and privacy reasons, this case study focused only on a selected group of HVAC installations that includes: two AHUs, three boilers, and three double pumps. The facility is equipped with a building automation system (BAS) that monitors and controls the different installations in the facility.

### 5.2. Data and Model Characteristics

According to the guideline defined previously in the framework, data sources were identified. The available sources are presented below.

**Figure 5:**
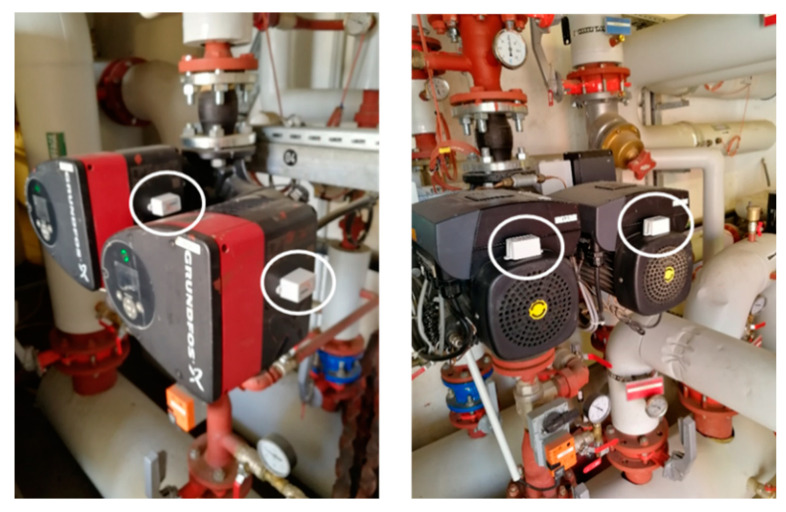
A photo of a vibration device installed on a double pump.

- The building automation system (BAS) was connected to a web server. Each installation is monitored via the BAS through one or multiple variables such as temperature, energy consumption, water consumption, and air or water flow rate, etc. A report from the BAS is uploaded every hour. The report contains the date and the time, the name of the variable and its value at that time.
- An extract from the CMMS that contains a part of the maintenance record.
- Vibration device: an IoT device was installed on the surface of the equipment; it is used to collect the vibration measurements on the installation. The data reported by the device are the acceleration measures on the three axis (*x*, *y* and *z*-axis), the frequency of vibration of the three axis, a binary variable (ON/OFF, which detects if the machine is enabled) and the temperature in the surface of the equipment. An example is presented in Figure 5 that shows a vibration device attached to a double pump.
- Electric meter device: an IoT meter installed to collect the electric energy data which includes the following measures: electric current intensity and voltage as well as the temperature on the surface of the equipment.

The IoT devices were only attached to the surface of the installations so that no deterioration or harm happens to the installations. The IoT devices are connected to the internet via the Lora network [65]. They were programmed to upload the measures in a 1 h cycle. The data are uploaded and stored on the web platform of Objenious: a French company (By Bouygues Telecom) specialising in IoT solutions and development.

Table 1 presents the group of the HVAC installations used in this study and the IoT device attached to each installation. The vibration device was attached to each installation. Due to accessibility issues, the electric meter device was only attached to the two AHUs. 

**Figure 6:**
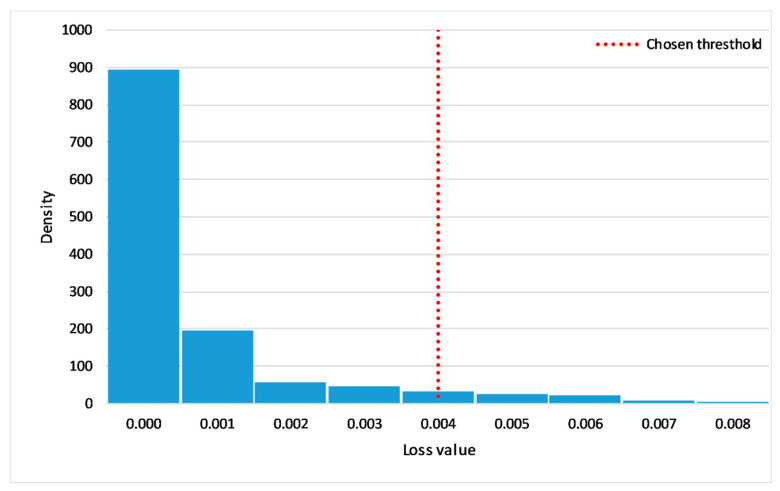
Distribution of the prediction error.

In order to build the training dataset, data were collected for a period of around 3 months. The predictive model used in this case study has the same architecture presented in Section 4.4.1. The root mean square error (RMSE) was used as an anomaly score (Section 4.4.2). Following this, the alert threshold was in accordance with the user to limit the number of alerts below an acceptable number. As a result of this process, the threshold was set to 0.0040, considering that the anomaly score (RMSE) varies from 0 to 0.0125 (dimensionless measure), as illustrated in Figure 6.

### 5.3. Results and Analysis

The model was tested for a period of 45 days (from 10 April 2020 to 25 May 2020). The results are presented in Table 2. There are three possibilities: (1) confirmed failure or true positive (the algorithm truly predicted a failure, then it is confirmed by the technician in the site). (2) Not confirmed failure or false positive (false alarm and no failure was reported in the site). (3) Failure not detected (the algorithm fails to report a failure). As shown in Table 2, four alerts were issued; two of them were confirmed as a true positive, and the other two were reported as false positive. During this period, a failure happened in “AHU 1” but the model failed to detect it. The true positive alerts were issued two days before they occurred on site.

This demonstrates that the algorithm can predict failures in advance. False positives are accepted since they can correspond to small anomalies that have not been reported as failures. However, the algorithm needs to be improved and to be tested on a bigger set of data.

**Figure 7:**
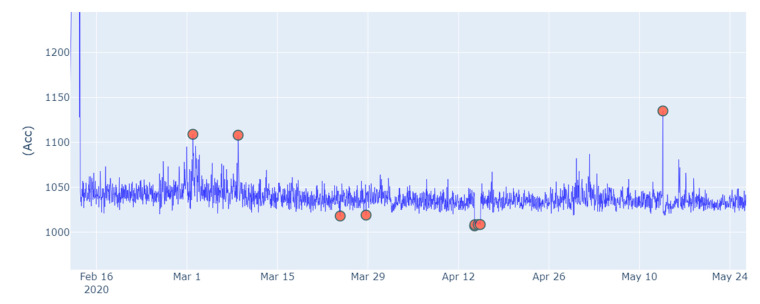
Projection of the anomalies detected in ‘Boiler 2’ on the vibration graph of the ‘Boiler 2’.

For instance, Figure 7 illustrates the anomalies detected in “Boiler 2”. The anomalies are projected on the vibration graph of “Boiler 2”, where the *x*-axis represents the time, and the *y*-axis represents the vibration (unit: $1\;\mathrm{mGal}\;=\;1\times 10^{-5}{m/s}^{2}$).

The first observation from this case study is the low number of alerts and failures during this period. This is due to the COVID-19 pandemic lockdown, which coincided with this period. Indeed, the sport facility was closed, and the HVAC installations were operating at their minimum regime, making failures less likely to happen.

One of the limitations of this case study is the small duration of data collection. This period does not take into consideration the changes in the HVAC operations related to change in seasons or the change in occupancy levels in the facility (big events). These changes in the regime are the cause of the majority of breakdowns and failures in the HVAC system according to the FM team in the facility. However, this trial phase was necessary and required by the FM team to give first feedback and results about the model.

In accordance with the user, data and feedback will be collected for a period of one year to take into consideration the different changes. This aims to give an accurate evaluation of the model and helps to improve the model for further implementation.

## 6. Discussion and Conclusions

The primary purpose of this study was to develop a generic framework for the predictive maintenance for buildings. For this, this study incorporated a literature review and face-to-face interviews with FM experts. Before designing the framework, identifying data sources in the building environment was necessary since data are the essence of the approach. A not exhaustive list was defined containing the most frequent sources in the buildings. Then the framework was organised into five steps: (1) data collection, (2) data processing, (3) model development, (4) fault notification, and (5) model improvement. A case study was addressed to demonstrate the implementation of the framework. This case study has multiple limitations, mainly related to the duration of the study and the small size of the collected data. However, this case study illustrates the process to implement the framework. It also revealed that the topic of predictive maintenance for building installations presents multiple opportunities as well as multiple challenges. Below, we reiterate some observations from the case study.

- Data between diversity and scarcity: data in the building environment are diverse in terms of sources and in terms of nature. They are generated from the human activity indoors, from the diverse installations in the building (mechanical, electrical, electronic etc.), and from the building itself. However, the majority of data are not collected and not stored. Moreover, unlike other industries, there is a lack of open databases containing building data, except some databases mainly focusing on building energy consumption [66]. As a result, building predictive maintenance has become a hard and a costly task.
- Return on investment: predictive maintenance strategy offers the facility manager the possibility to take early action to prevent failures, which improves the lifespan of the installations and improves the comfort of the inhabitants. However, the implementation of predictive maintenance may take a significant time to build an effective model. This presents an important barrier for the facility managers to invest in solutions that can take a significant time before it starts getting profitable.
- Each building is unique: unlike other industries, such as the manufacturing industry, where multiple installations are the same, each building has a different use, different architecture, and different occupancy. Thus, two same AHUs are not the same anymore once they are installed in different buildings. This unicity of buildings presents multiple opportunities and a wide market for predictive maintenance; moreover, it reveals several challenges to developing effective and affordable solutions.

In order to address the limitations of the case study, data will be collected for a one-year period to improve the training dataset. Further work will also focus on implementing the framework in different buildings to test the limits of scaling over multiple buildings.

## Figures and Tables

**Table 1:** The HVAC installations and the attached IoT devices.

Quantity	Installation	Attached IoT Devices
2	AHU	Vibration device Electric meter device
3	Boilers	Vibration device
3	Double pump	Vibration device

**Table 2:** Summary of faults alerts during the test period.

Installation	Alert Date	Feedback
Boiler 2	14 April 2020	Confirmed Failure
Boiler 2	15 April 2020	Confirmed Failure
AHU 2	25 April 020	Not Confirmed
Boiler 2	12 May 2020	Not Confirmed
AHU 1	Not detected	Failure not detected

## Data Availability

The data presented in this study are available on request from the corresponding author. The data are not publicly available due to confidentiality agreement with the company.
